# Epidemiological and Molecular Surveillance of Multiresistant *Citrobacter freundii* Complex in a Tertiary Care Hospital: A Retrospective Cohort Study

**DOI:** 10.1093/infdis/jiag066

**Published:** 2026-02-04

**Authors:** Pérince Fonton, Roberto Sierra, Romain Martischang, Aude Nguyen, Abdessalam Cherkaoui, Diego O Andrey, Stephan Harbarth

**Affiliations:** Infection Control Program, Geneva University Hospitals and Faculty of Medicine, WHO Collaborating Center, Geneva, Switzerland; Department of Microbiology and Molecular Medicine, Faculty of Medicine, University of Geneva, Geneva, Switzerland; Department of Microbiology and Molecular Medicine, Faculty of Medicine, University of Geneva, Geneva, Switzerland; Infectious Diseases Division, Department of Medicine, Geneva University Hospitals and Faculty of Medicine, Geneva, Switzerland; Division of Laboratory Medicine, Diagnostics Department, Geneva University Hospitals and Faculty of Medicine, Geneva, Switzerland; Infectious Diseases Division, Department of Medicine, Geneva University Hospitals and Faculty of Medicine, Geneva, Switzerland; Infectious Diseases Division, Department of Medicine, Geneva University Hospitals and Faculty of Medicine, Geneva, Switzerland; Division of Laboratory Medicine, Diagnostics Department, Geneva University Hospitals and Faculty of Medicine, Geneva, Switzerland; Department of Microbiology and Molecular Medicine, Faculty of Medicine, University of Geneva, Geneva, Switzerland; Infectious Diseases Division, Department of Medicine, Geneva University Hospitals and Faculty of Medicine, Geneva, Switzerland; Division of Laboratory Medicine, Diagnostics Department, Geneva University Hospitals and Faculty of Medicine, Geneva, Switzerland; Infection Control Program, Geneva University Hospitals and Faculty of Medicine, WHO Collaborating Center, Geneva, Switzerland

**Keywords:** molecular epidemiology, *Citrobacter freundii*, multidrug resistance, plasmid transmission, hospital acquired

## Abstract

**Background:**

*Citrobacter freundii* is an emerging nosocomial pathogen, yet its transmission dynamics remain poorly characterized.

**Methods:**

We conducted a retrospective genomic and epidemiological study to investigate the transmission chains of extended-spectrum beta-lactamase (ESBL) and carbapenemase-producing *C. freundii* collected at Geneva University Hospitals during a 6-year period (2017–2022). Whole-genome sequencing (WGS) using Nanopore long-read sequencing, MLST and plasmid typing, and resistome profiling were performed. Transmission events were defined based on genetic relatedness, plasmid similarity and patient trajectories.

**Results:**

A total of 103 cases of ESBL and carbapenemase-producing *C. freundii* were identified, of which 79% were hospital-acquired, 72% were isolated from rectal swabs, and bacteremia occurred in 6 patients. Among 90 isolates available for WGS, 73% were confirmed as *C. freundii*, whereas the remaining belonged to other *Citrobacter* species. A high genetic diversity was observed among *C. freundii* sensu stricto, with 29 distinct sequence types, including predominant ST114, ST98, and ST22. We identified 10 clusters involving multiple *Citrobacter* species among 35 patients, with a median cluster duration of 103 weeks (IQR, 65–138). Transmission chain analysis revealed that 37% of patients were involved in putative clonal transmission and 21% in putative plasmid-mediated dissemination events, particularly in abdominal surgery, geriatrics and septic orthopedics, often involving *C. freundii* harboring *bla*_CTX-M-15_, *bla*_OXA-48/CTX-M-14b_, and *bla*_OXA-181_ on IncHI2-IncHI2A, IncM1, and IncX3 plasmids, respectively.

**Conclusions:**

Our study highlights the role of *C. freundii* as a vector of both clonal and plasmid-mediated, nosocomial transmission of antimicrobial resistance. It emphasizes the need to include *C. freundii* in genomic and epidemiological surveillance to detect its silent spread.

*Citrobacter freundii* is an emerging multidrug-resistant nosocomial pathogen [1], increasing patient morbidity and mortality [2, 3]. A recent systematic review highlighted that *C. freundii* is increasingly implicated in nosocomial outbreaks, emphasizing its role as a driver of antimicrobial resistance (AMR) in hospitals settings [4]. Furthermore, carbapenem-resistant *C. freundii* has been identified as an emerging healthcare-related problem, since it represents a significant reservoir of multiple plasmids and resistance genes [5–7]. These plasmids facilitate horizontal gene transfer and promote the nosocomial dissemination of multiresistant strains that may occur over extended periods [8, 9].

Since its initial description in 1932, the genus *Citrobacter* has undergone multiple taxonomic rearrangements and now comprises 19 recognized species based on genomic relatedness [10]. Species within this genus are closely related and often lack accurate identification by MALDI-TOF, with the exception of other well-characterized species such as *Citrobacter koseri* [11]. In terms of antibiotic resistance, we distinguish *Citrobacter* spp. such as *C. koseri* and *Citrobacter amalonaticus*, characterized by low-level penicinillase production, from *C. freundii*, possessing a chromosomally encoded Ambler class C cephalosporinase [12, 13]. Despite its increasing clinical significance, there remains a notable gap in epidemiological and genetic understanding of this emerging pathogen.

Using molecular epidemiology, we aimed to investigate, through long-read whole-genome sequencing (WGS), the genomic characteristics with putative clonal and plasmid transmission chains of extended-spectrum beta-lactamase-producing (ESBL-P) and carbapenemase-producing (CP) *C. freundii* isolates identified in patients at Geneva University Hospitals (HUG).

## METHODS

### Study Design

This is a retrospective cohort study including (1) prospectively collected surveillance data and (2) genomic sequences of ESBL-P and CP *C. freundii* isolates retrieved from clinical specimens or screening swabs sampled from 1 January 2017 through 31 December 2022 at HUG. Only the first isolate per patient was included. In the case of simultaneous cocarriage of different ESBL-P and CP *C. freundii* strains, both isolates were analyzed.

### Study Setting, Screening Practices, and Infection Control Strategies

HUG is a large tertiary care hospital located in Geneva, Switzerland. Institution-wide targeted admission screening is performed on patients at high risk for ESBL-P and CP Enterobacterales carriage [14]. Additionally, universal weekly screening is performed in the adult intensive care unit, transplant, septic orthopedics and hematological wards. All bloodstream infections (BSIs) are investigated by the infection control team [15]. Hospital onset BSIs are recorded in a dedicated database [16, 17]. Our institution's infection control recommendations advocate contact precautions for all identified cases of ESBL-P and CP *C. freundii* [18]. Single bedrooms are mandated for patients carrying CP *C. freundii.*

### Definitions

While ESBL-P and CP cases were included based on both phenotypic and genotypic criteria, for the analysis, resistance was defined solely based on WGS-detected resistance genes, a method validated for reliably identifying both ESBL and carbapenemase determinants in Enterobacterales [19, 20] . Infection/colonization was classified as nosocomial (hospital-acquired) if (1) detected >48 hours after admission, or (2) if the patient had a prior hospitalization within 10 days before the current admission. The time at risk was defined as days from admission to the first positive sample of *C. freundii*.

A clonal transmission event was defined as a group of patients harboring isolates with identical multilocus sequence types (MLST), belonging to the same cluster by cgMLST analysis, and carrying the same ESBL-P or CP resistance gene. For cgMLST, we applied a cluster cutoff of ≤20 loci differences to infer possible relatedness. This threshold, based on previous publications on Enterobacterales including *Citrobacter* spp., is slightly higher than the ≤10-loci threshold previously proposed [21, 22], in order to capture events over extended time periods and those potentially linked to long-term environmental reservoirs. A putative plasmid-mediated transmission event was characterized by isolates expressing the same resistance gene on the same plasmid replicon type (Inc), as defined by Marimuthu et al. [23], and subsequently showing close relatedness based on core plasmid backbone analysis, without applying a fixed cutoff value. A possible transmission event (clonal or plasmid) was subsequently compared with patients’ spatiotemporal trajectories/ward admissions at HUG. Epidemiological direct transmission was defined as patient stays occurring simultaneously in the same unit, whereas indirect transmission, possibly mediated through an environmental reservoir [9, 24] or unobserved intermediate cases, was defined as stays in the same unit without temporal overlap. All cases that did not meet the 2 aforementioned criteria were classified as unlinked [23]. Medical procedures and devices (eg, endoscopes) were not specifically investigated.

### Data Collection

We integrated several distinct data sources: the surveillance databases of nosocomial multidrug-resistant bacteria (containing detailed clinical and epidemiological information, species, resistance mechanism and encoding genes, sampling site), electronic health records for additional epidemiological data, and our biobank for microbiological and genomic details from all ESBL-P and CP *Citrobacter* spp. Routine surveillance includes data on age, sex, department/units of hospitalization, length of stay, time from admission to the first positive culture, sampling site, and resistance mechanisms.

### Bacterial Isolates, Identification, and Resistance Genes Detection

Blood cultures were performed using a Bactec FX instrument (Becton, Dickinson and Company). Urine cultures (Becton Dickinson, *Vacutainer*™ Urine Collection) followed by aerobic growth on CHROMID*®*CPS ELITE agar (bioMérieux) and rectal surveillance swabs (eSwab™, Copan) plated on selective media including CHROMID*®*ESBL (bioMérieux) and *C*HROMID*®*OXA*-*48 (bioMérieux) were processed at the bacteriology laboratory at HUG, on a fully automated system (WASPLab®, Copan) coupled to artificial intelligence [25, 26].

Colonies were then identified by MALDI-TOF/MS using a cutoff of 2 for species identification (MBT Compass 4.1, Bruker Daltonics). Isolates were further tested for ESBL production using the double disk synergy test method and carbapenemase production by the Eazyplex SuperBug CRE system (Amplex Biosystems) [18].

### WGS and Bioinformatic Analysis

Genomic DNA was extracted from a bacterial pellet using the HMW DNA Extraction Kit (Promega) and quantified using Qubit Fluorometer (ThermoFisher Scientific). The libraries were generated using the ONT Sequencing by ligation kit V14 (SQK-NBD114.24, Oxford Nanopore Technologies), then loaded into an R10.4.1 flow cell and sequenced in a GridION sequencer using the super-accurate basecalling model. The resulting fastq files were used for assemblies with Flye (v.2.9) and polished with Medaka (v.2.0). Genomic species were identified using average nucleotide identity (ANI) as implemented in FastANI (v1.34). Assembled genomes were used for identification of chromosomal multilocus sequence types (MLST), plasmid incompatibility (Inc) groups and acquired antibiotic resistance genes using the available scheme for *C. freundii* and curated databases in ResFinder and PlasmidFinder from the Center for Genomic Epidemiology (Technical University of Denmark). Plasmid annotation and visualization were performed with Proksee.ca. Core genome MLST (cgMLST) for *C. freundii* [22], and whole-genome MLST (wgMLST) analyses for other *Citrobacter* spp. were performed using SeqSphere + (v10.0.5, Ridom) with ad hoc schemes defined for each *Citrobacter* species, using unique reference genomes from NCBI Genome database: *C. amalonaticus* (NZ_CP083651), *C. braakii* (NZ_CP045771), *C. portucalensis* (NZ_CP092466), *C. pasteurii* (NZ_CP077262), *C. europaeus* (NZ_CP148084), and *C. werkmanii* (NZ_CP044101). The resulting wgMLST schemes comprised 4375, 4190, 4598, 4270, 4399, and 4325 loci for *C. amalonaticus*, *C. braakii*, *C. portucalensis*, *C. pasteurii*, *C. europaeus,* and *C. werkmanii,* respectively. Core plasmid backbones were obtained by reciprocal blast analyses of plasmids recovered in this study together with reference plasmids of the same replicon type from previous studies [27–31]. Pairwise comparisons of core plasmid sequences were then performed using ANI to generate a backbone similarity matrix. Plasmid allelic distance-based clustering analysis was subsequently conducted using pyMLST and visualized as minimum spanning trees [32]. The allelic composition of each core plasmid, as defined by pyMLST, was additionally represented as heatmaps.

### Statistical Analysis

Categorical variables were reported as frequencies and proportions. Differences in patient characteristics were compared using Pearson's χ^2^ test. The incidence of ESBL-P and CP *C. freundii* cases was calculated per 10 000 patient-days. Linear regression lines were drawn for time trends. All statistical analyses were performed with R statistical software version 4.3.1 (R Foundation).

### Ethics

Data used in this study were collected retrospectively from routine surveillance only; therefore, informed consent was not required according to the Swiss law for research on human beings.

## RESULTS

### Study Population and Epidemiological Description

**Figure 1.**
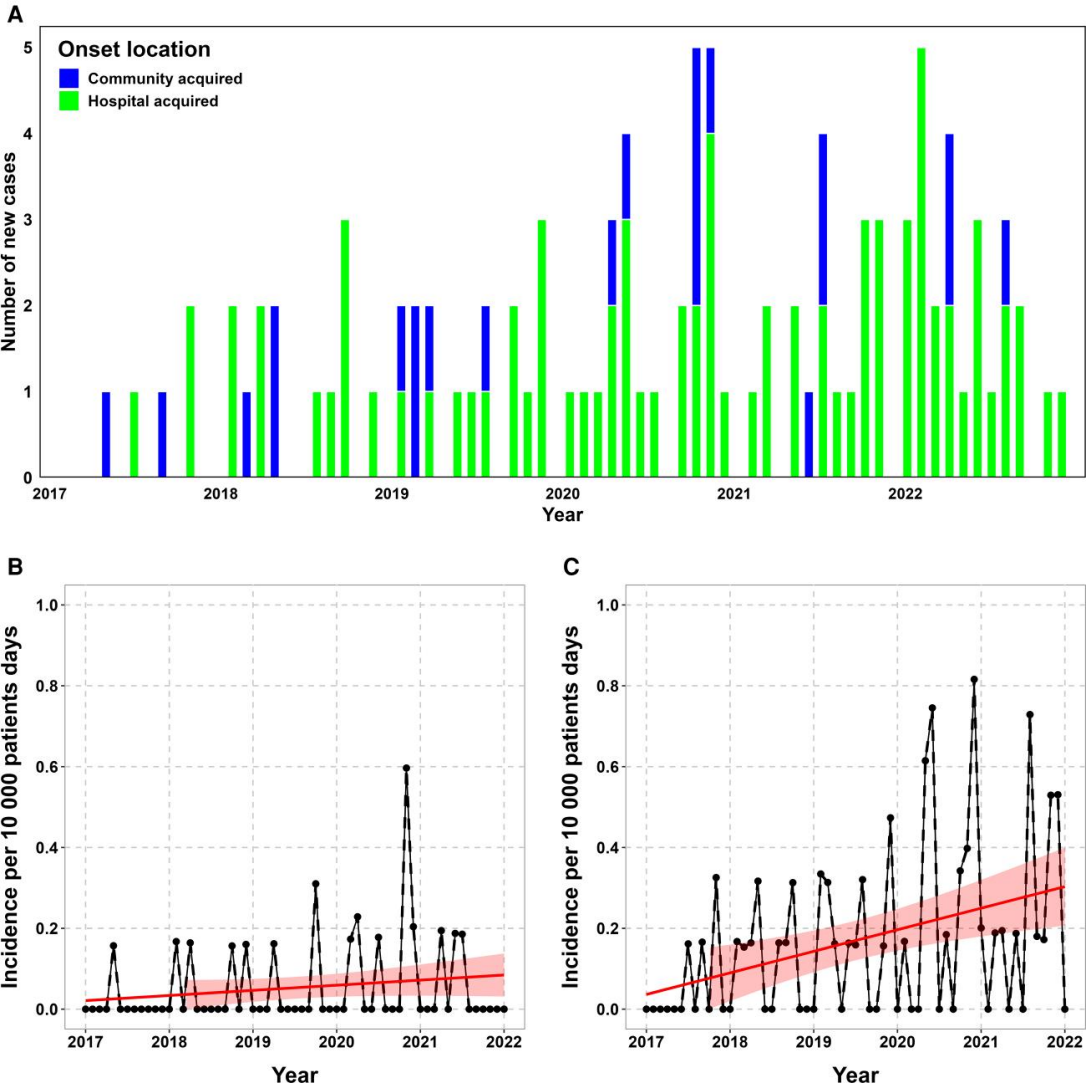
Temporal dynamics of ESBL- and carbapenemase-producing *C. freundii* at Geneva University Hospitals (2017–2022). *A*, Epidemic curve of newly identified cases of ESBL-P and/or CP *C. freundii* at HUG, January 2017 to December 2022. *B and C*, Incidence of ESBL and/or CP *C. freundii* cases per 10 000 patients-days between January 2017 and December 2022, stratified by (*B*) clinical isolates, and (*C*) rectal screening swabs. The red line shows the linear trend over time.

From January 2017 to December 2022, 103 incident cases were identified as infected or colonized with multiresistant *C. freundii* (Figure 1*A*). We observed an increase in its incidence from 2017 to 2022, both in clinical and surveillance cultures (Figure 1*B* and *C*). Important patient features are presented in Table 1, stratified by place of acquisition. Among identified cases, 58.2% were older than 65 years and 60.1% were male. Most cases had intestinal carriage only (71.8%) and were hospital-acquired (78.6%). Among clinical specimens, *C. freundii* was mostly isolated in urines (20.4%); BSI occurred in 6 patients. Most patients were hospitalized in rehabilitation/geriatrics and the surgical department at the time of positive sample; the majority of community-acquired cases were detected by surveillance swabs performed on admission.

**Table 1.** Characteristics of Patients Carrying Multiresistant *C. freundii* Isolates (Incident Cases, HUG, 2017–2022)

		All Incident *C. freundii* Cases (n = 103)	Community-acquired (n = 22)	Hospital-acquired (n = 81)	
		n (%)	n (%)	n (%)	*P* Value
Age (y)	<18	6 (5.8)	3 (13.6)	3 (3.7)	.14
…	18–64	37 (36.0)	9 (41.0)	28 (34.5)	
…	≥65	60 (58.2)	10 (45.4)	50 (61.7)	
Sex	Male	62 (60.1)	13 (59.0)	49 (60.5)	>.9
Time at risk (d) median, (IQR)	…	…	…	22 (8–56)	…
Length of stay after date of identification (d) median, (IQR)	…	19 (5–42)	9 (2–24)	21 (6–51)	.018
Sample type	Rectal swab	74 (71.8)	17 (77.3)	57 (70.4)	.4
	Urine	21 (20.4)	3 (13.6)	18 (22.2)	
	Blood	6 (5.8)	1 (4.5)	5 (6.2)	
	Others	2 (2.0)	1 (4.5)	1 (1.2)	
Hospital sector^a^	Rehabilitation and geriatrics	25 (24.3)	2 (9.0)	23 (28.4)	.03
	Surgery	24 (23.3)	4 (18.1)	20 (24.7)	
	ICU and ER	16 (15.5)	9 (41.0)	7 (8.6)	
	Medicine	15 (14.5)	1 (4.5)	14 (17.2)	
	Oncology	13 (12.6)	2 (9.0)	11 (13.6)	
	Pediatrics	6 (5.8)	3 (13.6)	3 (3.7)	
	Others	4 (3.9)	1 (4.5)	3 (3.7)	

Abbreviations: ER, emergency room; ICU, intensive care unit; IQR, interquartile range.

^a^At the time of the first positive sample included.

### Genomic Analysis and AMR Gene Determination

Of the 103 initial cases of ESBL-P and/or CP *C. freundii* isolates, 90 isolates were available for genomic analysis. WGS revealed that among those 90 isolates initially identified as *C. freundii* complex through MALDI-TOF, 66 (73.3%) were confirmed as *C. freundii* sensu stricto, the remaining 24 included *C. portucalensis* (n = 8), *C. werkmanii* (n = 5), *C. europaeus* (n = 4), *C. pasteurii* (n = 3)*, C. braakii,* and *C. amalonaticus* (n = 2 each), based on ANI analysis.

**Figure 2.**
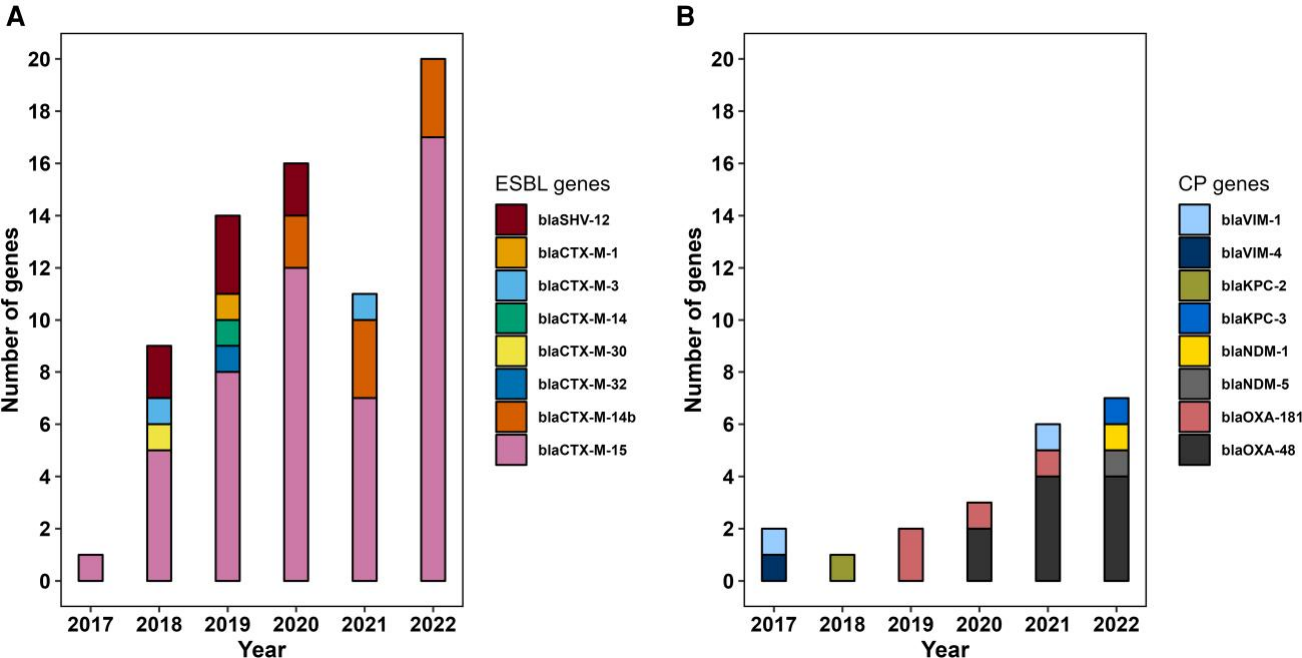
Temporal trends in antimicrobial resistance gene burden in *Citrobacter* spp. at Geneva University Hospitals (2017–2022). Annual number of antimicrobial resistance genes detected per isolate, stratified by (*A*) ESBL genes and (*B*) carbapenemase genes.

A high degree of genetic diversity was observed among the 90 isolates based on their MLST scheme. We found 29 distinct sequence types (STs) among *C. freundii* isolates, the most prevalent clones included ST114 (n = 10), ST98 and ST22 (n = 7 each), ST216 (n = 6), and ST19 (n = 5; Supplementary Table 1). Among the 90 isolates sequenced, 53 (58.9%) were exclusively ESBL-P, 16 (17.8%) coproduced both ESBL and a carbapenemase, and 3 isolates (3.3%) were CP only. In 18 isolates (20%), neither CTX-M nor SHV-containing ESBL were identified, but other β-lactamases, including several non carbapenemase OXA-genes, were found (Supplementary Table 1). The predominant ESBL genes identified were *bla*_CTX-M-15_ (n = 50), *bla*_CTX-M-14b_ (n = 8), and *bla_S_*_HV-12_ (n = 7; Figure 2*A*). Among carbapenemase-encoding genes, *bla*_OXA-48_ (n = 10) and *bla*_OXA-181_ (n = 4) were the most frequently detected (Figure 2*B*). AMR gene analysis also revealed that all species except *C. amalonaticus* and *C. europaeus* harbored the chromosomal encoded AmpC-type β-lactamase *bla*_CMY_, predominantly *bla*_CMY-48_ (n = 34), followed by *bla*_CMY-109_ (n = 7), and *bla*_CMY-117_/*bla*_CMY-152_/*bla*_CMY-98_ (n = 5 each). In *C. europaeus,* the AmpC-type β-lactamase *bla*_CFE-1_ was detected, whereas in the 2 *C. amalonaticus* isolates, the chromosomal inducible class A β-lactamase gene *bla*_CdiA_ was present (blastx 99.3% identity).

**Figure 3.**
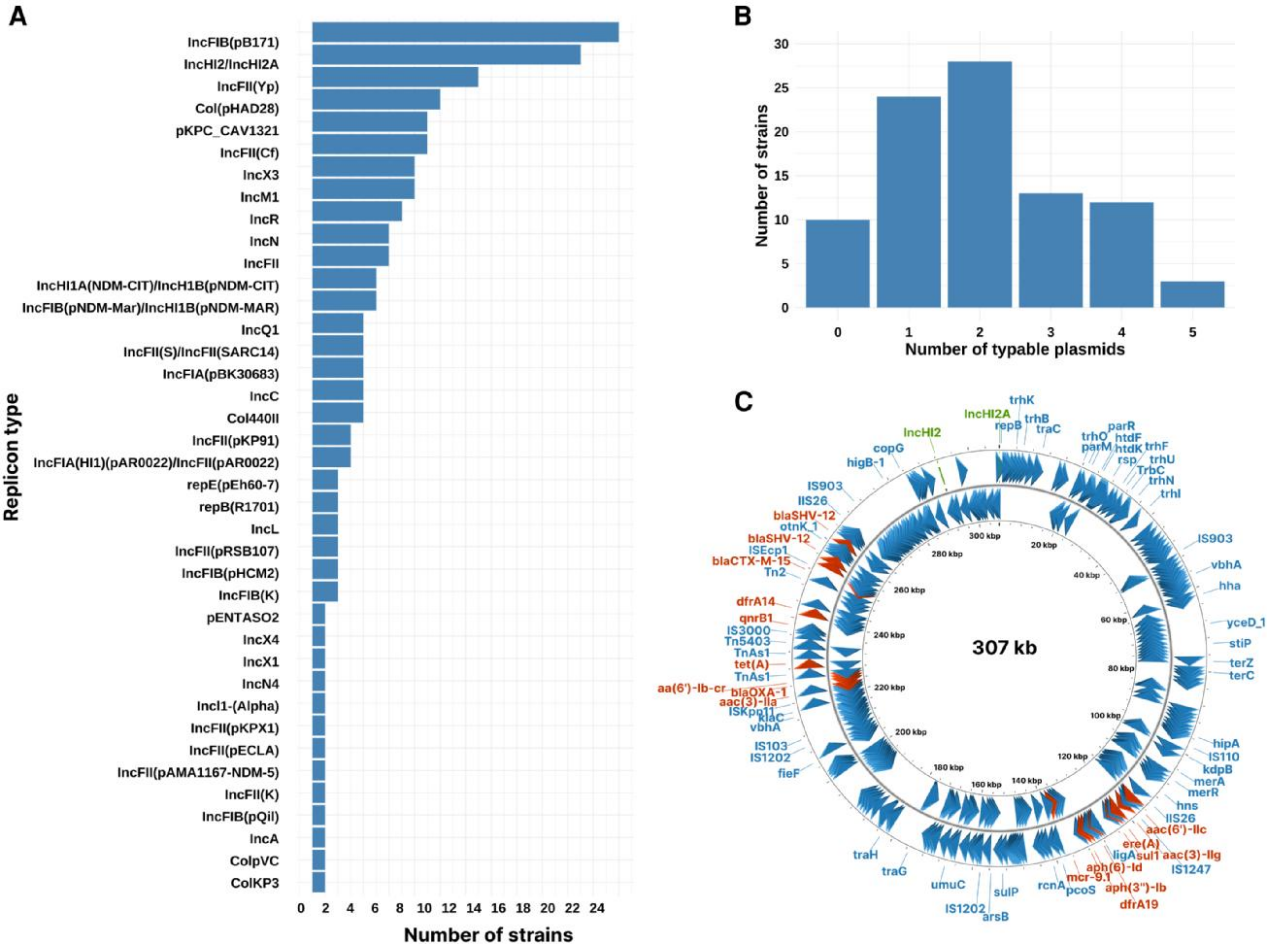
Plasmid description of multiresistant *Citrobacter* spp. at Geneva University Hospitals (2017–2022). *A*, Frequency of plasmid replicon types. *B*, Distribution of the number of distinct plasmids per isolate. *C*, Circular map of an IncHI2-IncHI2A plasmid carrying *bla*_CTX-M-15_ and mcr-9.1 identified in the current study.

The genomic analysis also revealed a highly diverse plasmid population in *C. freundii*. We identified 39 types of plasmids based on replicon typing (Figure 3*A* and Supplementary Table 1), including IncHI2-IncHI2A for *bla*_CTX-M-15_ and for carbapenemase plasmid *bla*_OXA-181_-IncX3 and *bla*_OXA-48_-IncM1. In 10 isolates, no plasmid was detected, with resistance genes located on the chromosome. The majority of strains (n = 56) carried between 2 and 5 plasmids (Figure 3*B*). Beyond β-lactamases, the colistin resistance gene *mcr*-9.1 was detected in 4 ESBL-P *C. freundii* isolates from distinct genetic backgrounds, 3 of which carried an IncHI2-IncHI2A plasmid (Figure 3*C* and Supplementary Table 1). The genomic localization of AMR genes is shown in Supplementary Figure 1, stratified by replicon type or chromosomal location.

### Cluster Analysis and Transmission Dynamics

**Figure 4.**
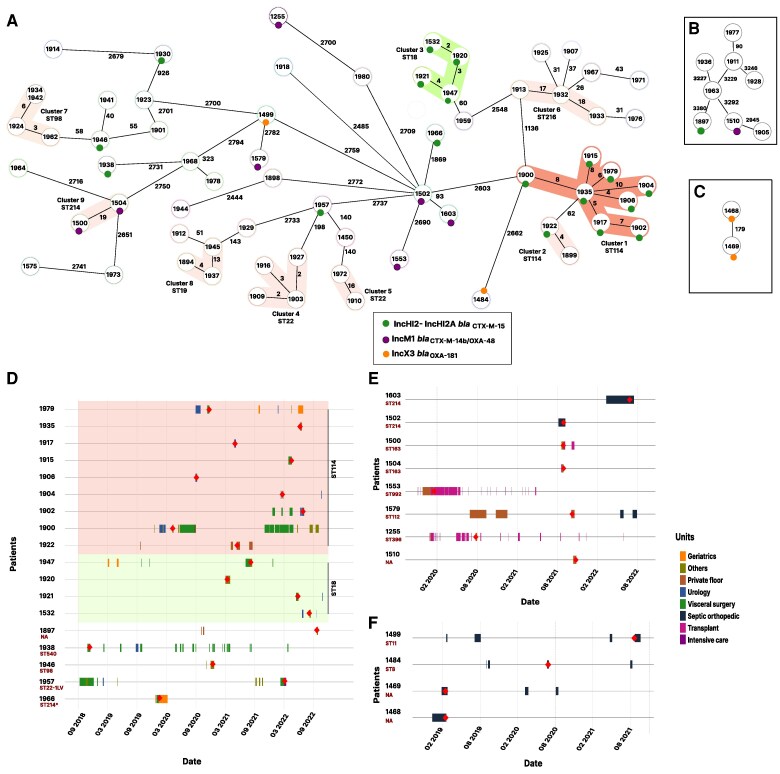
Clonal and plasmid-mediated transmission of *Citrobacter* spp. and associated patient trajectories at Geneva University Hospitals (2017–2022). Minimum spanning trees reconstructed from (*A*) cgMLST for *C. freundii* sensu stricto and (*B* and *C*) wgMLST for other *Citrobacter* spp., depicting genetic clusters associated with putative clonal and plasmid-mediated transmission events. *A*, 66 *C. freundii* isolates analyzed using 3250 cgMLST loci, (*B*) 8 *C. portucalensis* isolates analyzed using 4598 wgMLST loci, and (*C*) 2 *C. amalonaticus* isolates analyzed using 4375 wgMLST loci. Green circles represent *bla*_CTX-M-15_ carried on IncHI2-IncHI2A plasmids, purple circles represent the *bla*_CTX-M-14b/OXA-48_ carried on IncM1 plasmids, and orange circles represent *bla*_OXA-181_ carried on IncX3 plasmids. Only selected putative plasmid transmission events are shown. (*D-F*) Patient trajectories within HUG for isolates harboring (*D*) *bla*_CTX-M-15_ located on IncHI2-IncHI2A plasmids (light pink: ST114 clusters 1 and 2; and light green ST18 cluster 3), (*E*) *bla*_OXA-48_ and *bla*_CTX-M-14b_ on IncM1 plasmids, and (*F*) *bla*_OXA-181_ on IncX3 plasmids.

cgMLST analysis identified 10 clusters involving multiple *Citrobacter* species and 35 patients, with a median cluster duration of 103 weeks (interquartile range, 65–138) (Figure 4 and Supplementary Figure 2). Two clusters corresponded to *C. freundii* ST114, comprising 8 and 2 patients respectively, all carrying *bla*_CTX-M-15_ (Figure 4*A* and *D*). Additional clonal clusters included isolates belonging to ST22 and ST18, also harboring *bla*_CTX-M-15_ on IncHI2-IncHI2A plasmids. Notably, this plasmid type was also detected in 5 genetically unrelated isolates, suggesting a putative plasmid-mediated transmission route (Figure 4*A* and *D*). Another plasmid-mediated cluster was suspected by colocalization of *bla*_CTX-M-14b_ and *bla*_OXA-48_ on IncM1 plasmids, detected in 7 *C. freundii* and 1 *C. portucalensis* isolates across different STs (Figure 4*A* and *E*). Similarly, we identified 2 unrelated *C. freundii* and 2 *C. amalonaticus* isolates, all carrying *bla*_OXA-181_ located on IncX3 plasmids, suggestive of horizontal transmission via shared mobile elements (Figure 4*A*, *C*, and *F*).

In total, 37% (33/90) of cases were attributed to putative clonal transmission, 21% (19/90) to plasmid-mediated transmission and 42% (38/90) remained unlinked. By combining cgMLST data with spatiotemporal mapping of patient movements (Figure 4*D*–*F*), we identified both clonal and horizontal transmission events within and across hospital wards, across extended time periods. Transmission events were particularly obvious in abdominal surgery and geriatric units, where a long-term plasmid transmission chain involving IncHI2-IncHI2A plasmids carrying *bla*_CTX-M-15_ spanned over 4 years and involved 18 patients, mainly associated with ST114 and ST18. Another major plasmid cluster involved 8 patients across the septic orthopedics and private units, between February 2020 and August 2022, all harboring IncM1 plasmids cocarrying *bla_C_*_TX-M-14b_ and *bla*_OXA-48_ across diverse *Citrobacter* spp. (Figure 4*E*). In the septic orthopedic unit, we identified 4 patients carrying *bla*_OXA-181_ isolates (including *C. freundii* and *C. amalonaticus*) sharing an IncX3 plasmid, despite belonging to different STs (Figure 4*F*). Several additional, smaller clusters were also suspected through integration of molecular and epidemiological data.

**Figure 5.**
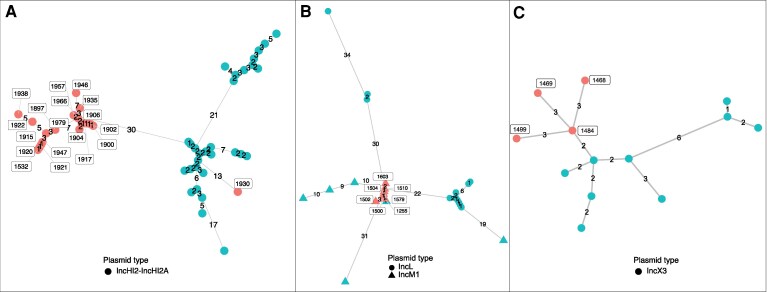
Plasmid allelic distance-based clustering analysis. Minimum spanning trees of core plasmid backbones for (*A*) IncHI2-IncHI2A, including 30 reference plasmids [27] (*B*) IncM1/IncL, including 17 reference plasmids [28], and (*C*) IncX3, including 9 reference plasmids [30, 31]. Clustering was performed using core plasmid allele matrices specific to each replicon background. Red nodes correspond to plasmids identified in the current study, and green nodes represent plasmids from previous studies.

Putative plasmid transmissions were further investigated by assessing plasmid relatedness using core plasmid backbone reconstruction and ANIs (Supplementary Figures 3–5), followed by allelic distance-based clustering analysis (Figure 5). All but one IncHI2-IncHI2A plasmid were found to be closely related (with the exception of the plasmid identified in strain 1930). All IncM1-*bla*_OXA-48_ plasmids exhibited close relatedness and were separated in terms of allelic differences to most published comparative plasmids of IncM1 and related IncL. All IncX3 plasmids were likewise found to be closely related. Overall, these molecular analyses support the majority of the putative plasmid transmission events identified for these 3 plasmid-mediated clusters.

## DISCUSSION

The principal findings of this study are: (1) the nosocomial incidence of ESBL-P and CP *C. freundii* is increasing, with most cases representing asymptomatic colonization; (2) clonal and plasmid-mediated transmission chains involving multiple *Citrobacter* species were detected in specific units over extended time periods; (3) there is complex and often silent dissemination of resistance genes within the hospital ecosystem; and (4) routine MALDI-TOF-based identification of *Citrobacter* spp. leads to inaccurate species-level assignments and supports the current laboratory practice of reporting at the complex level. Overall, this study provides a comprehensive picture of clinical, phenotypic and genetic AMR information of a complex opportunistic pathogen.

The predominance of nosocomial acquisition among multiresistant *Citrobacter* spp. is consistent with Indian and South Korean studies reporting hospital-acquired *Citrobacter* spp. in 94.6% and 78.1% of cases, respectively [33, 34]. The increasing institutional incidence of multiresistant *C. freundii* observed is also consistent with national trends, as a recent Swiss study reported a sustained annual rise in *Citrobacter* spp. since 2010 [35].

We identified 27% of non-*C. freundii* isolates after genome sequencing. Furthermore, among the 87 *C. freundii* isolates classified as ESBL producers based on phenotypic testing, only 77% were genetically confirmed, with phenotypic misclassification likely due to the presence of chromosomal AmpC type β-lactamase and other β-lactamases (non-ESBL/CP). These findings corroborate earlier concerns regarding the taxonomic intricacy of the *C. freundii* group, and the challenges in accurately characterizing AMR based on phenotypic data alone, thereby underscoring the importance of genomic surveillance [11].

The major clones identified in this study (ST114, ST22, ST98, and ST18), are consistent with previously reported *C. freundii* lineages described in Finland, France, and Belgium [6, 8, 21], and a global study including 67 countries [36]. We documented the long-term persistence and silent dissemination of *C. freundii* ST22 carrying *bla*_CTX-M-15_ between 2017 and 2022. A similar prolonged circulation of ST22 carrying *bla*_OXA-48_ has been reported from Belgium, involving both clinical isolates and hospital plumbing systems over multiple years [6].

We documented the circulation of similar IncHI2-IncHI2A plasmids carrying *bla*_CTX-M-15_ across genetically unrelated *Citrobacter* spp. These specific plasmids, frequently harboring multiple resistance genes, have been described as widespread AMR drivers among various Enterobacterales including *Citrobacter* spp. [37–39]. This plasmid backbone is self-transmissible and has been implicated in the global dissemination of New Delhi Metallo-β-lactamases (NDM) [40, 41]. We observed the circulation of an IncX3 plasmid carrying *bla*_OXA-181_ across 4 unrelated *Citrobacter* isolates, also consistent with a recent genomic study in Germany [42]. Additionally, the presence of *bla*_OXA-48_ and *bla*_CTX-M-14b_ on IncM1 plasmids in *Citrobacter* spp. has been recently documented among nosocomial *Klebsiella pneumoniae* strains in Wales [43]. Similar IncM1 plasmids coharboring these resistance genes have also been identified within our institution, showing high sequence similarity and interspecies conjugative potential [28]. These observations support the role of IncM1 plasmids as efficient vectors of multidrug resistance across diverse Enterobacterales in healthcare settings.

It is noteworthy that clonal and putative plasmid-mediated transmission events involving multiple *Citrobacter* spp. were detected in 37% and 21% of isolates, respectively. For patients without overlapping hospital stays, the observed transmission patterns suggest a potential contribution of environmental reservoirs or indirect transmission pathways. The importance of plasmidic transmission is consistent with the findings of Marimuthu et al. [23], who demonstrated putative clonal and plasmid-mediated transmission events of *C. freundii* in 22% and 47.5% of patients, respectively ([23], with additional personal communication). This higher proportion of plasmid-mediated transmission events is likely to be related to the inclusion of all Enterobacterales and environmental isolates in that study from Singapore.

In our study, we initially identified putative plasmid transmission events, which were subsequently evaluated across 3 plasmid clusters using core plasmid backbone analysis in combination with epidemiological data. This integrative approach supported the majority of the proposed plasmid-mediated transmission events. Comparison with previously published plasmids revealed clear allelic differences between plasmids identified in our hospital and published IncHI2-IncHI2A plasmids [27]. Similar findings were observed for OXA-48-carrying IncM1 plasmids, which diverged from IncM1 plasmids reported from other hospitals as well as from IncL OXA-48 plasmids, except for 1 reference plasmid (CP083077) that clustered with our hospital plasmids; notably, this plasmid was also isolated elsewhere in Switzerland, consistent with regional circulation. Finally, OXA-181-carrying IncX3 plasmids, although displaying limited genetic diversity, clustered closely and were all identified within the same ward. Nevertheless, this approach may possibly overestimate the number of plasmid transmission events, particularly in the absence of validated distance cutoffs for defining plasmid clusters.

The units affected by multiresistant *Citrobacter* spp. circulation in our study included septic orthopedics, abdominal surgery, geriatrics and private wards, likely reflecting local epidemiological patterns, as well as patient- and unit-specific exposures. A recent systematic review [4] similarly reported the frequent involvement of surgical wards in *Citrobacter* spp. outbreaks,. Furthermore, a recent environmental study at our institution observed multiresistant *Citrobacter* spp. in 6% of 137 sinks sampled between July 2021 and February 2022 (including 7 surgical wards) [44]. Several silent transmission cycles of multiresistant Enterobacterales have previously been documented in our geriatric and long-term care facilities [45], prone to sustained transmission risk.

This study provides one of the most comprehensive molecular and epidemiological assessments of multiresistant *C. freundii* transmission in a tertiary care setting over a 6-year period, integrating long-read WGS with detailed spatiotemporal patient-level data. However, several limitations should be acknowledged. First, as a single-center study, the findings may not be fully generalized to other healthcare institutions. However, a recent study from Germany described similar findings and hidden CP-*Citrobacter* transmission chains [46]. Second, the absence of systematic screening at hospital admission and discharge may have resulted in under-detection of asymptomatic carriers and missed transmission events. Third, due to the retrospective design, environmental reservoirs were not systematically investigated. However, hospital plumbing and wastewater systems likely served as sources for clonal and plasmid-mediated transmission, as previously demonstrated [6]. Moreover, our epidemiological analyses were limited to geo-temporal associations, preventing assessment of indirect transmission routes such as those involving healthcare workers or medical devices. Fourth, this study only focused on *C. freundii*, likely underestimating plasmid-mediated transmission events, notably those involving other Enterobacterales. Fifth, some of the community-acquired cases may reflect previous contamination in external healthcare facilities. Finally, the threshold used to define genetic relatedness in our study was selected by expert consensus, guided by prior evidence. Currently, no evidence-based mutation rates or molecular epidemiological benchmarks exist to define a standardized genomic threshold for *C. freundii*. Consequently, our selected threshold was adapted from prior investigations [47, 48].

## CONCLUSIONS

By integrating genomic and epidemiological data, this study highlights the significant yet often under-recognized role of *C. freundii* as an active vector of AMR, with evidence of both clonal and plasmid-mediated transmission. We further identified the involvement of specific high-risk hospital units in the local dissemination of multiresistant *C. freundii*, possibly influenced by patient, and ward-level factors. These findings highlight the importance of including this pathogen in future clinical and environmental surveillance efforts to improve our understanding of AMR transmission dynamics in healthcare settings.

## Supplementary Material

jiag066_Supplementary_Data

## Supplementary Data

Supplementary materials are available at *The Journal of Infectious Diseases* online (http://jid.oxfordjournals.org/). Supplementary materials consist of data provided by the author that are published to benefit the reader. The posted materials are not copyedited. The contents of all supplementary data are the sole responsibility of the authors. Questions or messages regarding errors should be addressed to the author.
