# Supplementary material for: Epidemiological and Molecular Surveillance of Multiresistant Citrobacter freundii Complex in a Tertiary Care Hospital: A Retrospective Cohort Study
Source: J Infect Dis. 2026 Feb 4;233(5):e1181–92. doi: 10.1093/infdis/jiag066 (PMC13175611; doi:10.1093/infdis/jiag066)
Supplement: jiag066_Supplementary_Data [file jiag066_supplementary_data.zip › Supplementary material.docx]

**Epidemiological and molecular surveillance of multiresistant *Citrobacter freundii* complex in a tertiary care hospital: A retrospective cohort study**

Running title: Genomic epidemiology of *C. freundii*

Pérince Fonton^1, a^, Roberto Sierra^2,3,4,a^, Romain Martischang^3^, Aude Nguyen^3^, Abdessalam Cherkaoui^4^, Diego O. Andrey^2,3,4,b,*^, Stephan Harbarth^1,b,*^

Affiliations:

1. Infection Control Program, Geneva University Hospitals and Faculty of Medicine, WHO Collaborating Center, Rue Gabrielle-Perret-Gentil 4, 1205, Geneva, Switzerland.
2. Department of Microbiology and Molecular Medicine, Faculty of Medicine, University of Geneva, Switzerland, Rue Michel Servet 1, 1206, Geneva, Switzerland.
3. Infectious Diseases Division, Department of Medicine, Geneva University Hospitals, Geneva, Switzerland, Rue Gabrielle-Perret-Gentil 4, 1205, Geneva, Switzerland.
4. Division of Laboratory Medicine, Diagnostics Department, Geneva University Hospitals and Faculty of Medicine, Rue Gabrielle-Perret-Gentil 4, 1205, Geneva, Switzerland.

* Corresponding authors: stephan.harbarth@hug.ch, [diego.andrey@hug.ch](mailto:diego.andrey@hug.ch)

a, b: Authors contributed equally to the work.

**Figure S1**: Bubble chart depicting the localization and abundance of resistance genes associated with Inc plasmid type and chromosome. The bubble size is proportional to resistance gene count.

**Figure S2**: Clonal and plasmid-mediated transmission of *Citrobacter* spp. Minimum spanning trees (MST) reconstructed from wgMLST data of *Citrobacter* spp. isolates depicting genetic clusters of putative clonal transmission and plasmid-mediated transmission events, for five *C. werkmanii* isolates using 4325 genes (A), four *C.* *europaeus* using 4399 genes (B), two *C. braakii* using 4190 genes (C) and three *C. pasteurii* using 4270 loci (D).

**Figure S3**. IncHI2-IncHI2A core plasmid analysis. Heat-maps (A) illustrating the pairwise comparison of average nucleotide identities (ANI) and (B) allelic composition of plasmid backbones for IncHI2-IncHI2A plasmids including 30 plasmids from Macesic et al., 2021.

**Figure S4**. IncM1/IncL core plasmid analysis. Heat-maps (A) illustrating the pairwise comparison of ANI and (B) allelic composition of plasmid backbones for IncM1/IncL plasmids including 17 plasmids from Sierra et al., 2025, and one clonal plasmid of Swiss origin (CP083077) from Findlay et al., 2022.

**Figure S5**. IncX3 core plasmid analysis. Heat-maps (A) illustrating the pairwise comparison of ANI and (B) allelic composition of plasmid backbones for IncX3 plasmids including nine plasmids from Venditti et al.,2017 and Yu et al.,2022.

**References**

1. Macesic N, Blakeway LV, Stewart JD, et al. Silent spread of mobile colistin resistance gene mcr-9.1 on IncHI2 ‘superplasmids’ in clinical carbapenem-resistant Enterobacterales. *Clinical Microbiology and Infection*. 2021;27(12):1856.e7-1856.e13. doi:10.1016/j.cmi.2021.04.020

2. Sierra R, Roch M, Prados J, et al. Horizontal transmission of a multidrug-resistant IncM1 plasmid harbouring *bla*OXA-48 and *bla*CTX-M-14b among patient microbiotas. *J Antimicrob Chemother*. Published online July 18, 2025: dkaf228. doi:10.1093/jac/dkaf228

3. Findlay J, Perreten V, Poirel L, Nordmann P. Molecular analysis of OXA-48-producing *Escherichia coli* in Switzerland from 2019 to 2020. *Eur J Clin Microbiol Infect Dis*. 2022;41(11):1355-1360. doi:10.1007/s10096-022-04493-6

4. Venditti C, Fortini D, Villa L, et al. Circulation of *bla*KPC-3-Carrying IncX3 Plasmids among *Citrobacter freundii* Isolates in an Italian Hospital. *Antimicrob Agents Chemother*. 2017;61(8):e00505-17. doi:10.1128/AAC.00505-17

5. Yu Z, Zhang Z, Shi L, et al. In silico characterization of IncX3 plasmids carrying bla OXA-181 in Enterobacterales. *Front Cell Infect Microbiol*. 2022; 12:988236. doi:10.3389/fcimb.2022.988236

**Alt text for supplement Figure:**

Figure S1 Alt Text: Distribution of antimicrobial resistance genes across plasmid replicon types and chromosome.

Figure S2 Alt Text: Minimum spanning trees reconstructed from wgMLST of *C.* *werkmanii, C. europaeus*, *C. braakii,* and *C. pasteurii*.

Figure S3 Alt Text: Heatmaps of IncHI2-IncHI2A plasmids showing (A) pairwise ANI and (B) allelic composition of core backbone genes.

Figure S4 Alt Text: Heatmaps of IncM1/IncL plasmids showing (A) pairwise ANI and (B) allelic composition of core backbone genes.

Figure S5 Alt Text: Heatmaps of IncX3 plasmids showing (A) pairwise ANI and (B) allelic composition of core backbone genes.
